# Sphenoid Sinus Mucosal Flap after Transsphenoidal Surgery—A Systematic Review

**DOI:** 10.3390/medicina60020282

**Published:** 2024-02-06

**Authors:** Piotr Sumislawski, Martyna Piotrowska, Jan Regelsberger, Jörg Flitsch, Roman Rotermund

**Affiliations:** 1 Department of Neurosurgery, Faculty of Medicine, University Hospital Carl Gustav Carus, Technische Universität Dresden, Fetscherstrasse 74, 01307 Dresden, Germany; piotr.sumislawski@gmail.com; 2 Faculty of Medicine and Dentistry, Pomeranian Medical University, Al. Powstańców Wielkopolskich 72, 70-111 Szczecin, Poland; martynap221@o2.pl; 3 Department of Neurosurgery, Diako Krankenhaus Flensburg, 24939 Flensburg, Germany; regelsbergerja@diako.de; 4 Department of Neurosurgery, University Medical Center Hamburg-Eppendorf, Martinistr. 52, 20246 Hamburg, Germany; flitsch@uke.de

**Keywords:** CSF leak, sphenoid mucosa, sphenoid sinus mucosal flap, pituitary surgery, skull base reconstruction, sellar reconstruction, transsphenoidal surgery

## Abstract

*Background and Objectives:* Skull base reconstruction is a crucial step during transsphenoidal surgery. Sphenoid mucosa is a mucosal membrane located in the sphenoid sinus. Preservation and lateral shifting of sphenoid mucosa as sphenoid mucosal flap (SMF) during the transsphenoidal exposure of the sella may be important for later closure. This is the first systematic review to evaluate the utility of sphenoid mucosal flap for sellar reconstruction after transsphenoidal surgery. *Materials and Methods:* A systematic literature search was performed in January 2023: Cochrane, EMBASE, PubMed, Scopus, and Web of Science. The following keywords and their combinations were used: “sphenoid mucosa”, “sphenoid sinus mucosa”, “sphenoid mucosal flap”, “sphenoid sinus mucosal flap”. From a total number of 749 records, 10 articles involving 1671 patients were included in our systematic review. *Results:* Sphenoid sinus mucosa used to be applied for sellar reconstruction as either a vascularized pedicled flap or as a free flap. Three different types of mucosal flaps, an intersinus septal flap, a superiorly based flap and an inferiorly based flap, were described in the literature. Total SMF covering compared to partial or no SMF covering in sellar floor reconstruction resulted in fewer postoperative CSF leaks (*p* = 0.008) and a shorter duration of the postoperative lumbar drain (*p* = 0.003), if applied. Total or partial SMF resulted in fewer local complications (*p* = 0.012), such as fat graft necrosis, bone graft necrosis, sinusitis or fungal infection, in contrast to no SMF implementation. *Conclusions:* SMF seems to be an effective technique for skull base reconstruction after transsphenoidal surgery, as it can reduce the usage of avascular grafts such as fat along with the incidence of local complications, such as fat graft necrosis, bone graft necrosis, sinusitis and fungal infection, or it may improve the sinonasal quality of life by maintaining favorable wound healing through vascular flap and promote the normalization of the sphenoid sinus posterior wall. Further clinical studies evaluating sphenoid mucosal flap preservation and application in combination with other techniques, particularly for higher-grade CSF leaks, are required.

## 1. Introduction

Sellar reconstruction is a crucial step during transsphenoidal surgery and may be associated with various complications, such as CSF leak, meningitis, pituitary abscess, rhinosinusitis, sinonasal mucocele or pyocele [1,2,3,4]. This procedure remains technically challenging, and the success rate depends on the defect size, intraoperative grade of CSF leakage, along with the selected operative techniques [5]. Various reconstruction techniques, such as avascular grafts (composite septal cartilage graft, muscle graft, fat graft or fascia lata graft), vascular flaps (nasoseptal mucosal flap, middle turbinate flap or anterior lateral nasal wall flap), artificial or bovine/equine dural substitutes (polydioxanone foil, Duragen, polytetrafluoroethylene, Duraform, Durafurm, TissuDura), lumbal drainage, hemostatic agents (oxidative cellulose, gelatine-thrombin matrix, Surgicel, Gelfoam, TachoComb, TachoSil, Greenplast, Evicel and other fibrin sealants) or combined techniques have been described [6,7,8,9,10,11,12,13,14,15,16,17,18,19,20]. 

The sphenoid sinus is air-filled space within sphenoid bone and lined with mucosal membrane commonly known as sphenoid mucosa. Preservation and lateral shifting of the sphenoid mucosal flap during the transsphenoidal exposure of the sella may be important, as several studies reported the advantage of sphenoid mucosal flap (SMF) application for skull base reconstruction after transsphenoidal surgery [17,18,21,22,23,24,25,26,27,28]. 

This is the first systematic review to evaluate the utility of SMF for skull base reconstruction after transsphenoidal surgery.

## 2. Methods

### 2.1. Search Strategy

**Figure 1:**
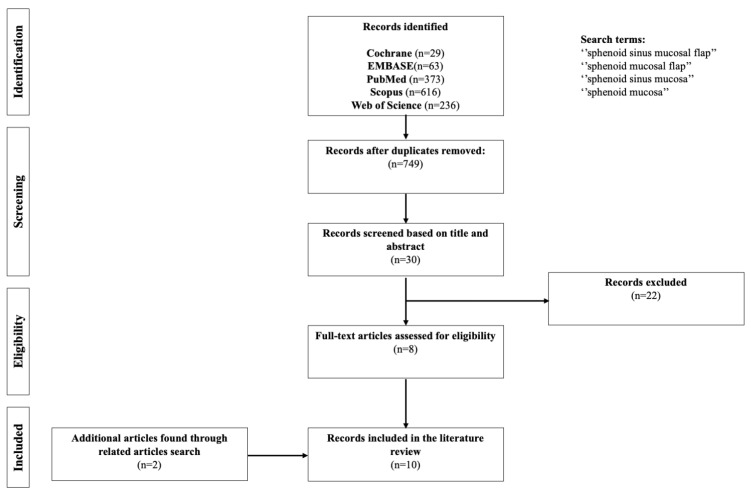
ENTREQ flow diagram for selected studies.

A systematic literature review based on Cochrane, EMBASE, PubMed, Scopus, and Web of Science databases was performed in May 2023 according to ENTREQ guidelines (see Reporting guideline checklist and Figure 1) by two independent reviewers (P.S. and M.P.) [29].

The following keywords and their combinations were used: “sphenoid mucosa”, “sphenoid sinus mucosa”, “sphenoid mucosal flap”, “sphenoid sinus mucosal flap”. Duplicate articles were excluded. The study was not registered in any systematic review database.

### 2.2. Study Selection

Eligibility criteria were: (1) original articles until 5 May 2023; (2) English only; (3) application of sphenoid mucosal flap after transsphenoidal surgery. 

### 2.3. Quality Appraisal

Quality appraisal using Critical Appraisal Skills Program (CASP) guidelines was conducted for all potentially relevant studies by two reviewers (P.S. and M.P.) [30]. Each selected study was appraised for quality and internal validity according to the CASP checklist (see CASP Checklist) for qualitative research. The CASP checklist contains 10 questions to assess the quality of qualitative research. 

### 2.4. Data Extraction

Extracted data with an overview of included studies are presented in Table 1 and Table 2. For the organization of extracted data, a unified matrix was utilized to record specific characteristics of included studies. Extracted data comprised: reference details (author, year, title, journal/publisher), objectives or aims of the study, study design, ethics (how ethical issues were addressed), sampling methodology, sample size, indication for sellar reconstruction, anatomical aspects, description of operative technique, complications and outcome after surgery. All calculations were performed on Microsoft Excel (version 2019; Microsoft).

### 2.5. Data Synthesis

Thematic synthesis is a well-established analytical technique for qualitative research and commonly published according to ENTREQ reporting guidelines [29]. 

During readings of the studies, similar findings were coded into descriptive themes (see Literature quotations) within and across studies. The process of acquiring the descriptive themes from initial codes was inductive to assessing previously researched phenomena. Two reviewers were involved in the coding and analysis (P.S. and M.P.).

### 2.6. Classification of Sellar Defect

The CSF leak and extension of skull base defect were classified according to the Esposito-Kelly grading system [31]. 

## 3. Results

We identified 749 studies after removing duplicates and excluded 741 studies for the following reasons: (1) the title and/or abstract did not match selection criteria; (2) studies were irrelevant when applied to inclusion criteria. In addition, we included two studies after a related article search. As a result, we included 10 studies in the qualitative synthesis.

### 3.1. Operative Technique

**Figure 2:**
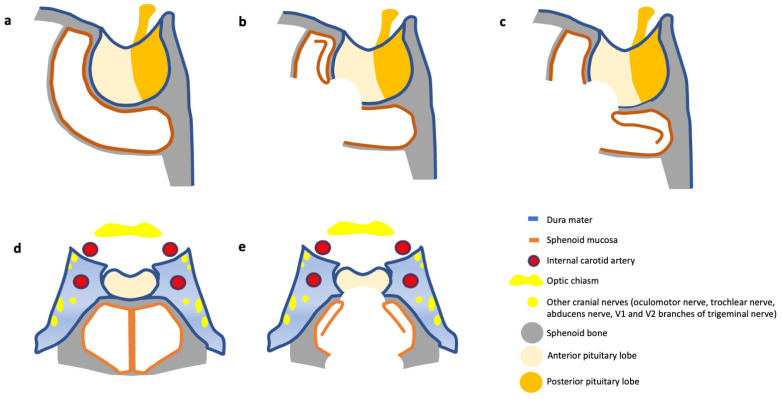
Illustration of different sphenoid mucosal flaps. (**a**,**d**) Normal preoperative sphenoid and sellar anatomy. (**b**) Superiorly and (**c**) inferiorly based mucosal flaps. (**e**) The most common intersinus septal mucosal flap.

Sphenoid sinus mucosa used to be applied for sellar reconstruction as either a well-vascularized pedicled flap or as a free flap, which can be subsequently sutured or stuck with fibrin glue covering the laceration or larger defect [21]. Three different types of sphenoid mucosal flaps, the intersinus septal mucosal flap, a superiorly based mucosal flap and an inferiorly based mucosal flap, were already described by Yoon and colleagues (see Figure 2) [28]. The intersinus septal mucosal flap was the most common and predominantly performed one after primary procedures. The superiorly based mucosal flap along with the inferiorly based mucosal flap were harvested mainly after multiple surgeries without an intersinus septum or in cases with multiple septums [28]. Furthermore, isolation of more than one flap and closure in a multi-layered fashion is possible [23]. SMF could be successfully implemented either by absence or small CSF leak as a stand-alone or in a combined technique for larger ones [17,18,21,22,23,24,25,26,27,28]. 

### 3.2. Advantages

**Figure 3:**
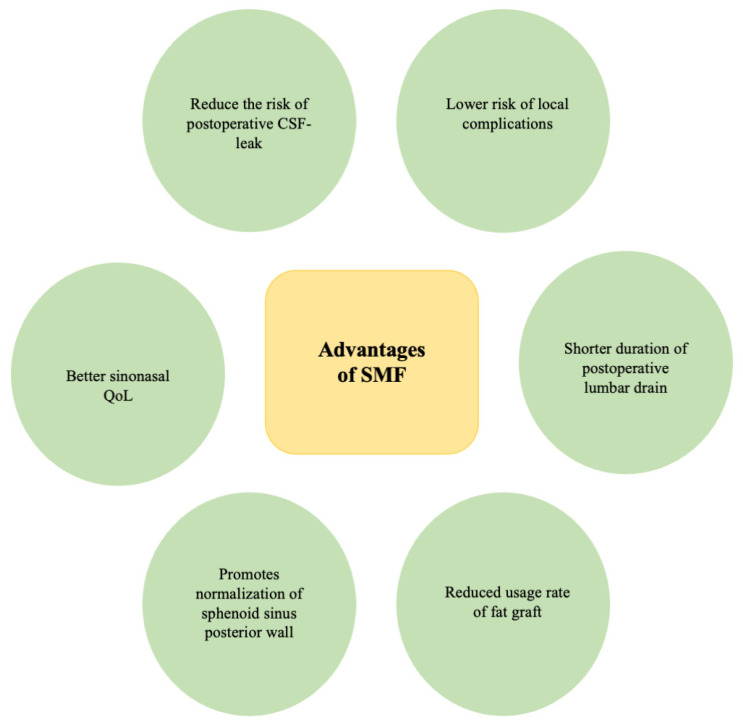
Advantages of SMF application.

When compared to partial or no SMF covering, total SMF covering in sellar floor reconstruction resulted in fewer postoperative CSF leaks (*p* = 0.008) and a shorter duration of the postoperative lumbar drain (*p* = 0.003) [28]. Total or partial SMF resulted in fewer local complications (*p* = 0.012), such as fat graft necrosis, bone graft necrosis, sinusitis or fungal infection, in contrast to no SMF implementation [28]. Application of SMF may also reduce the usage of fat grafts and the risk of associated necrosis [21,28]. SMF also promotes the normalization of the sphenoid sinus posterior wall [26]. Sellar reconstruction with SMF results in a better sinonasal QoL compared to NSF, as measured by the SNOT-22 Score for the first 6 weeks postoperatively (*p* < 0.05) [22]. The advantages of SMF are presented in Figure 3.

### 3.3. Limitations

**Figure 4:**
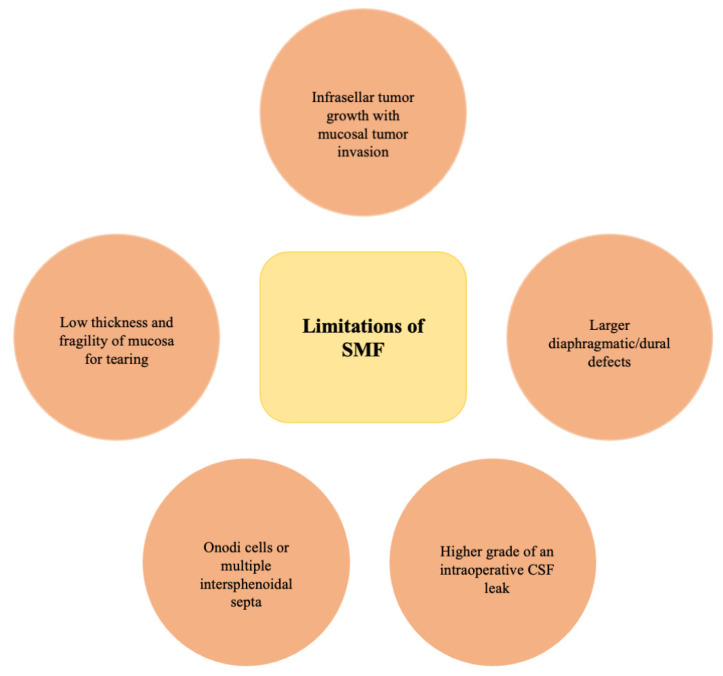
Limitations of SMF application.

The anatomy of sphenoid sinus and sphenoid mucosa is variable and may limit the application of SMF. Mucosal thickness may vary from being very dense and elastic, suitable for dura and bone defect reconstruction, to being skinny and fragile, susceptible to tearing by bony ridges of the sphenoid sinus or any surgical manipulation [21,23]. Anatomical variants such as Onodi cells or multiple intersphenoidal septa were described as limiting the usage of SMF [22]. Mucosal quality and its utility for sella repair can be limited by infections or infrasellar tumor invasion, where not only the sella turcica is being destructed but also sphenoid mucosa [21,22]. A worse mucosal quality with partial SMF covering is associated with a higher risk of CSF leak [28]. Postoperative irradiation may also interrupt the healing process, resulting in a delayed CSF leak [21]. Another issue is invagination of SMF after sellar reconstruction, which may be a result of increased intracranial pressure by sneezing, blowing the nose during the early healing process and/or an incompletely unfolded sphenoid mucosa during sellar floor reconstruction [27]. Clinically, it may be associated with a reduced improvement of postoperative headaches during early follow-up [27]. Limitations of SMF are summarized in Figure 4.

## 4. Discussion

### 4.1. Clinical Significance

Our systematic review presents the utility of SMF for sellar reconstruction after transsphenoidal surgery. SMF is a vascularized flap similar to the nasoseptal mucosal flap, middle turbinate flap or anterior lateral nasal wall flap. All these flaps present with a lower risk of necrosis, lower risk of CSF leak and support a better healing process compared to avascular grafts [32,33]. However the usage of NSF and MTF alters the physiological nasal or sphenoidal passage and may be associated with additional adverse effects, such as nasal fossa synechia, internal nasal valve failure, nasal dorsum collapse or septal perforation, while also lowering the sinonasal quality of life compared to SMF [21,22,34,35]. For small CSF leaks, both SMF and NSF have a comparable risk of CSF leak, and SMF may be applied as a stand-alone [22]. In the case of higher grades of CSF leak, SMF application alone may be insufficient, and the implementation of NSF along with other techniques such as multilayered closure may be necessary [21,22,25,36]. Avascular grafts, such as fat, muscle or fascia lata grafts, are usually implanted in the case of a lower risk or CSF rhinorrhoea and reveal a higher risk of local complications than SMF, such as necrosis or infections [28,36]. SMF application can reduce the usage rate of fat graft and the duration of lumbar drain for CSF leak [21,28]. Studies with sellar reconstruction using biomaterials (dural substitutes and hemostatic agents) disclosed a similar efficacy regarding CSF leaks and a more favorable side-effect profile compared to avascular grafts (fat or fascia lata graft) [14,15,16,19,20,37].

Preserved SMFs maintain the physiological restoration of the posterior sphenoid sinus wall and facilitate exploration through a potential reoperation [26,27]. Preoperative planning is not only essential for a surgical approach but also for preparing the strategy for sellar reconstruction. The presence of thin and fragile mucosa susceptible to tearing, Onodi cells, multiple intersphenoidal septa or pathological states, such as mucosal tumor invasion or previous infection, may restrict the application of SMF [21,22,28]. Invagination of SMF after sellar reconstruction remains a clinical issue [27]. To prevent this condition, the implementation of rigid materials resistant to physical pressure is recommended [27,37].

### 4.2. Limitations

Our review was limited by several aspects. Most of the included studies were non-comparative and investigated the application of SMF in combination with other techniques. As a consequence, the direct impact and significance of SMF for sellar floor reconstruction could not be quantified. Comparative studies were restricted by the small number of sample sizes and their retrospective nature. Therefore, correlations requiring prospective data, larger cohorts and more detailed information could not be conducted.

### 4.3. Perspectives

Future investigations should focus on a more comprehensive clinical and morphological analysis of sphenoid mucosa including different inter-patient anatomical variations to reveal the significance of its restoration and the potential limitation of SMF. To accomplish the highest grade of evidence, multicentric randomized controlled trials shall be designed.

## 5. Conclusions

The sphenoid mucosal flap (SMF) seems to be an effective technique for skull base reconstruction after transspenoidal surgery, as it can reduce the usage of avascular grafts, such as fat, along with the incidence of local complications, such as fat graft necrosis, bone graft necrosis, sinusitis or fungal infection, and may improve the sinonasal quality of life by maintaining favorable wound healing through the vascular flap and promote the normalization of the sphenoid sinus posterior wall. Further clinical studies evaluating sphenoid mucosal flap preservation and application in combination with other techniques, especially for higher-grade CSF leaks, are required.

## Figures and Tables

**Table 1:** Summary of all SMF studies’ characteristics.

Authors (Year)	N	Indication	Only SMF or Combined Technique	Technique		Successful Attempts (%)	
Amano et al. [21]	295	CSF-Leak Grade 1, 2 and 3	Only SMF/SSM (sphenoid sinus mucosa) patch or combined	SSM patching (partially with suturing), SMF or combination of SSM patching and SMF with/without fat or fascia graft		293 (99.3%)	
Castle-Kirszbaum et al. [22]	127	CSF-Leak Grade 0, 1	combined	Oxidized cellulose (37.8%), Gelatine sponge (23.6%), Autologous fat (8.7%), Polyethylene glycol hydrogel (18.1%), Fibrin sealant (44.9%), Gelatine-thrombin matrix (4.7%), Nasal tampons (37.8%), SMF		CSF-Leak Grade 0	98 (98%)
						CSF-Leak Grade 1	27 (100%)
Goel et al. [23]	42	N/A	combined	Gelfoam, bone chips, SMF		31 (73.8%)	
Goljo et al. [24]	1	CSF-Leak Grade 1	combined	Duragen, SMF, Surgicel, Gelfoam, Evicel		1 (100%)	
Hara et al. [25]	81	CSF-Leak Grade 1 and 2	combined	Gelfoam, fibrin glue, fat graft, SMF, dural sutures		CSF-Leak Grade 1	51 (100%)
						CSF-Leak Grade 2	30 (100%)
Jeong et al. [26]	37	CSF-Leak Grade 0	with/without SMF	SMF		13 with SMF	13 (100%)
						24 without SMF	24 (100%)
Kim et al. [27]	155	CSF-Leak Grade 0	only SMF	SMF		8 patients with SMF invagination	8 (100%)
						147 patients without SMF invagination	147 (100%)
Lee et al. [17]	827	CSF-Leak Grade 0, 1 and 2	combined	CSF-Leak Grade 0: oxidative cellulose, SMF, Greenplast		CSF-Leak Grade 0	609 (100%)
				CSF-Leak Grade 1: oxidative cellulose, Duraform or fat, SMF, DuraSeal		CSF-Leak Grade 1	129 (95.6%)
				CSF-Leak Grade 2: oxidative cellulose, Duraform or fat, SMF, DuraSeal		CSF-Leak Grade 2	76 (91.6%)
Park et al. [18]	38	CSF-Leak Grade 1 and 2a	combined	CSF-Leak Grade 1: Duraform, Surgicel, epidural septal bone, SMF, DuraSeal		CSF-Leak Grade 1	29 (100%)
				CSF-Leak Grade 2a: TachoComb, Duraform, Surgicel, epidural septal bone, SMF, DuraSeal		CSF-Leak Grade 2a	9 (100%)
Yoon et al. [28]	43 total SMF-covering	Both no intraoperative and intraoperative CSF Leak	combined	No CSF-Leak	Gelfoam, Fibrin sealant, SMF	37 (100%)	
				CSF-Leak	Gelfoam, Fibrin sealant, SMF, fat graft, lumbar drain	6 (100%)	
	25 partial or no SMF covering			No CSF-Leak	Gelfoam, Fibrin sealant, partial or no SMF	13 (92.9%)	
				CSF-Leak	Gelfoam, Fibrin sealant, partial or no SMF, fat graft, lumbar drain	6 (54.5%)	

N = number of patients in a study; Indication for sellar reconstruction was defined as a grade of CSF leak according to Esposito–Kelly grading system [31].

**Table 2:** Comparative studies regarding SMF.

Authors	Comparison	Results
Amano et al. [21]	SMF with or without fat graft	- reduced usage rate of fat graft after SMF (*p* = 0.00021)
Castle-Kirszbaum et al. [22]	SMF vs. NSF	- better sinonasal QoL after SMF compared to NSF up to 6 weeks postoperative (*p* < 0.05) - comparable risk of CSF leak between SMF and NSF, 98.4% vs. 100%, respectively
Jeong et al. [26]	with vs. without SMF	- significantly smaller postoperative volume in the SMF group than in the control group (*p* = 0.012) - smaller volume difference (postoperative minus preoperative) in the SMF group than in the control group (*p* = 0.046)
Kim et al. [27]	invagination of SMF vs. controls	- reduced improvement of postoperative headaches by invagination of SMF (*p* = 0.049)
Yoon et al. [28]	total SMF covering vs. partial or no SMF covering	- shorter duration of postoperative lumbar drain after SMF (*p* = 0.003) - lower risk of postoperative CSF leak (*p* = 0.008)
	total or partial SMF covering vs. no SMF covering	- lower risk of local complications: 1 fungal infection after total or partial SMF covering vs. 2 fat graft necrosis, 1 bone graft necrosis, 1 sinusitis and 1 fungal infection without SMF covering (*p* = 0.012)

## Data Availability

The data presented in this study are available on request from the corresponding author (accurately indicate status).
